# Associations of immunological features with COVID-19 severity: a systematic review and meta-analysis

**DOI:** 10.1186/s12879-021-06457-1

**Published:** 2021-08-03

**Authors:** Zhicheng Zhang, Guo Ai, Liping Chen, Shunfang Liu, Chen Gong, Xiaodong Zhu, Chunli Zhang, Hua Qin, Junhui Hu, Jinjin Huang

**Affiliations:** 1 Department of Gastroenterology, Tongji Hospital, Tongji Medical College, Huazhong University of Science and Technology, Wuhan, China; 2 Department of Molecular and Medical Pharmacology, David Geffen School of Medicine, University of California, Los Angeles (UCLA), Los Angeles, USA; 3 Department of Pediatrics, Tongji Hospital, Tongji Medical College, Huazhong University of Science and Technology, Wuhan, China; 4 Department of Oncology, Tongji Hospital, Tongji Medical College, Huazhong University of Science and Technology, Wuhan, China; 5 Department of Oncology, Huang Gang Central Hospital, Huanggang, China; 6 Department of Endocrinology, Huang Gang Central Hospital, Huanggang, China; 7 Department of Hematology, Tongji Hospital, Tongji Medical College, Huazhong University of Science and Technology, Wuhan, 430030 China

**Keywords:** COVID-19, Severity, Immune cells, Cytokines, Chemokines, meta-analysis

## Abstract

**Background:**

COVID-19 has spread widely worldwide, causing millions of deaths. We aim to explore the association of immunological features with COVID-19 severity.

**Methods:**

We conducted a meta-analysis to estimate mean difference (MD) of immune cells and cytokines levels with COVID-19 severity in PubMed, Web of Science, Scopus, the Cochrane Library and the grey literature.

**Results:**

A total of 21 studies with 2033 COVID-19 patients were included. Compared with mild cases, severe cases showed significantly lower levels of immune cells including CD3^+^ T cell (× 10^6^, MD, − 413.87; 95%CI, − 611.39 to − 216.34), CD4^+^ T cell (× 10^6^, MD, − 203.56; 95%CI, − 277.94 to − 129.18), CD8^+^ T cell (× 10^6^, MD, − 128.88; 95%CI, − 163.97 to − 93.79), B cell (× 10^6^/L; MD, − 23.87; 95%CI, − 43.97 to − 3.78) and NK cell (× 10^6^/L; MD, − 57.12; 95%CI, − 81.18 to − 33.06), and significantly higher levels of cytokines including TNF-α (pg/ml; MD, 0.34; 95%CI, 0.09 to 0.59), IL-5 (pg/ml; MD, 14.2; 95%CI, 3.99 to 24.4), IL-6 (pg/ml; MD, 13.07; 95%CI, 9.80 to 16.35), and IL-10 (pg/ml; MD, 2.04; 95%CI, 1.32 to 2.75), and significantly higher levels of chemokines as MCP-1 (SMD, 3.41; 95%CI, 2.42 to 4.40), IP-10 (SMD, 2.82; 95%CI, 1.20 to 4.45) and eotaxin (SMD, 1.55; 95%CI, 0.05 to 3.05). However, no significant difference was found in other indicators such as Treg cell (× 10^6^, MD, − 0.13; 95%CI, − 1.40 to 1.14), CD4^+^/CD8^+^ ratio (MD, 0.26; 95%CI, − 0.02 to 0.55), IFN-γ (pg/ml; MD, 0.26; 95%CI, − 0.05 to 0.56), IL-2 (pg/ml; MD, 0.05; 95%CI, − 0.49 to 0.60), IL-4 (pg/ml; MD, − 0.03; 95%CI, − 0.68 to 0.62), GM-CSF (SMD, 0.44; 95%CI, − 0.46 to 1.35), and RANTES (SMD, 0.94; 95%CI, − 2.88 to 4.75).

**Conclusion:**

Our meta-analysis revealed significantly lower levels of immune cells (CD3^+^ T, CD4^+^ T, CD8^+^ T, B and NK cells), higher levels of cytokines (TNF-α, IL-5, IL-6 and IL-10) and higher levels of chemokines (MCP-1, IP-10 and eotaxin) in severe cases in comparison to mild cases of COVID-19. Measurement of immunological features could help assess disease severity for effective triage of COVID-19 patients.

**Supplementary Information:**

The online version contains supplementary material available at 10.1186/s12879-021-06457-1.

## Background

Coronavirus disease 2019 (COVID-19) was caused by severe acute respiratory syndrome coronavirus 2 (SARS-CoV-2) infection, which has spread around the world [1]. Till September 09, 2020, the SARS-CoV-2 has infected over 27 million people and caused over 890,000 deaths [2]. The severity of COVID-19 may be strongly related to the immune status of patients, but this is poorly understood. Therefore, it is necessary to investigate the association of immunological features with COVID-19 severity, which may help identify immune markers for effective triage of COVID-19 patients.

Although some studies have focused on the association between immunologic features and COVID-19 severity, the conclusions remain controversial. Chen et al. found that the SARS-CoV-2 infection can decrease the counts of T lymphocytes, particularly CD4^+^ and CD8^+^ T cells [3]. Qin et al. showed that the cytokines of severe COVID-19 cases, such as tumor necrosis factor alpha (TNF-α), interleukin-5 (IL-5), IL-6 and IL-10, increased during the COVID-19 progression [4]. Sophie et al. demonstrated that the increased serum concentrations of IFN-γ inducible protein-10 (IP-10) and granulocyte-macrophage colony-stimulating factor (GM-CSF) were associated with day-28 mortality of COVID-19 patients [5]. However, other studies have revealed that CD4^+^ and CD8^+^ T cells [4], some cytokines (IL-5, IL-6 and IL-10) [6] and chemokines (GM-CSF and IP-10) [7] showed no significant differences between severe cases and mild cases. Thus, we presented a meta-analysis of 21 studies in order to assess the association between immune cells (CD3^+^ T, CD4^+^ T, CD8^+^ T, Treg, B and NK cells), cytokines (TNF-α, IFN-γ, IL-2, IL-4, IL-5, IL-6 and IL-10), chemokines (GM-CSF, RANTES, MCP-1, IP-10 and eotaxin) and COVID-19 severity respectively.

## Methods

### Search strategy

We performed a systematic literature search to identify relevant studies published up to September 09, 2020 in PubMed, Web of Science, Scopus, the Cochrane Library and the grey literature. The following combined search terms were used: (“Novel coronavirus” OR “Coronavirus disease 2019” OR “Coronavirus 2019” OR “nCoV-2019” OR “2019-nCoV” OR “COVID-19” OR “SARS-CoV-2”) and ((“CD3^+^ T” OR “CD4^+^ T” OR “CD8^+^ T” OR “CD4^+^/CD8^+^” OR “Treg” OR “B cell” OR “NK cell”) OR (“interferon gamma” OR “tumor necrosis factor alpha” OR “IL-2” OR “IL-4” OR “IL-5” OR “IL-6” OR “IL-10”) OR (“chemokine” OR “chemokines” OR “RANTES” OR “GM-CSF” OR “Eotaxin” OR “MCP-1” OR “IP-10”)).

### Study selection

Inclusion criteria of the study were as follows: 1) studies with data on immune cells (CD4^+^ T, CD8^+^ T, CD3^+^ T, CD4^+^/CD8^+^, Treg, B and NK cells), cytokines (IFN, TNF-α, IL-2, IL-4, IL-5, IL-6 and IL-10) and chemokines (GM-CSF, RANTES, MCP-1, IP-10 and eotaxin) with mean ± standard deviation (SD) or median (interquartile range, IQR); 2) patients could be grouped into severe cases and mild cases; and 3) studies with clear information on COVID-19 confirmation and included patients. The exclusion criteria were as follows: 1) studies without corresponding outcome of indicators; 2) studies without available full texts; 3) studies not published in English; 4) lack of mean ± SD or mean (IQR) of indicators; 5) reviews, editorials, case reports, and meta-analyses.

Two investigators developed the search strategy and one investigator conducted the primary systematic search for all studies meeting the predetermined inclusion criteria. The titles and abstracts of the retrieved articles were screened for duplication and relevance to the topic. A second investigator assessed study eligibility, quality assessment, and data extraction, for validity and consistency. Full-text reports of the identified citations were reviewed by both the primary and secondary investigators in order to select the final studies. All discrepancies were resolved by consensus, and if necessary, by consultation with the third investigator.

### Data extraction

The following data were extracted from each study: 1) the first author and year of publication; 2) study design; 3) the country where the study was conducted; 4) age; 5) sample size; 6) sex; 7) the levels of immune cells (CD4^+^ T, CD8^+^ T, CD3^+^ T, CD4^+^/CD8^+^, Treg, B and NK cells), and cytokines (IFN, TNF-α, IL-2, IL-4, IL-5, IL-6 and IL-10), and chemokines (GM-CSF, RANTES, MCP-1, IP-10 and eotaxin). Median (IQR) values were converted to mean ± SD using mathematical formulas as reported by Hozo et al. [8].

### Quality assessment

Quality assessments of the studies were carried out based on the Newcastle-Ottawa Scale (NOS). The total NOS score ≥ 7 indicated a good research quality of the included study.

### Data synthesis and analysis

Data entry and analysis were performed with Review Manager 5.3 (The Cochrane Collaboration, Oxford, England). Heterogeneity within each group of studies was assessed by Q test and *I*^*2*^ statistics. A random-effects model was used when *I*^*2*^ > 50% or *p <* 0.05 and a fixed-effects model was used when *I*^*2*^ ≤ 50% or *p* ≥ 0.05. For the continuous data, we calculated mean differences (MD) and 95% confidence intervals (CI) between severe cases and mild cases. To investigate the potential publication bias, we visually examined the funnel plots. To assess the robustness of the results, we performed sensitivity analyses by removing one study at a time.

## Results

### Search results and characteristics of included studies

**Fig. 1:**
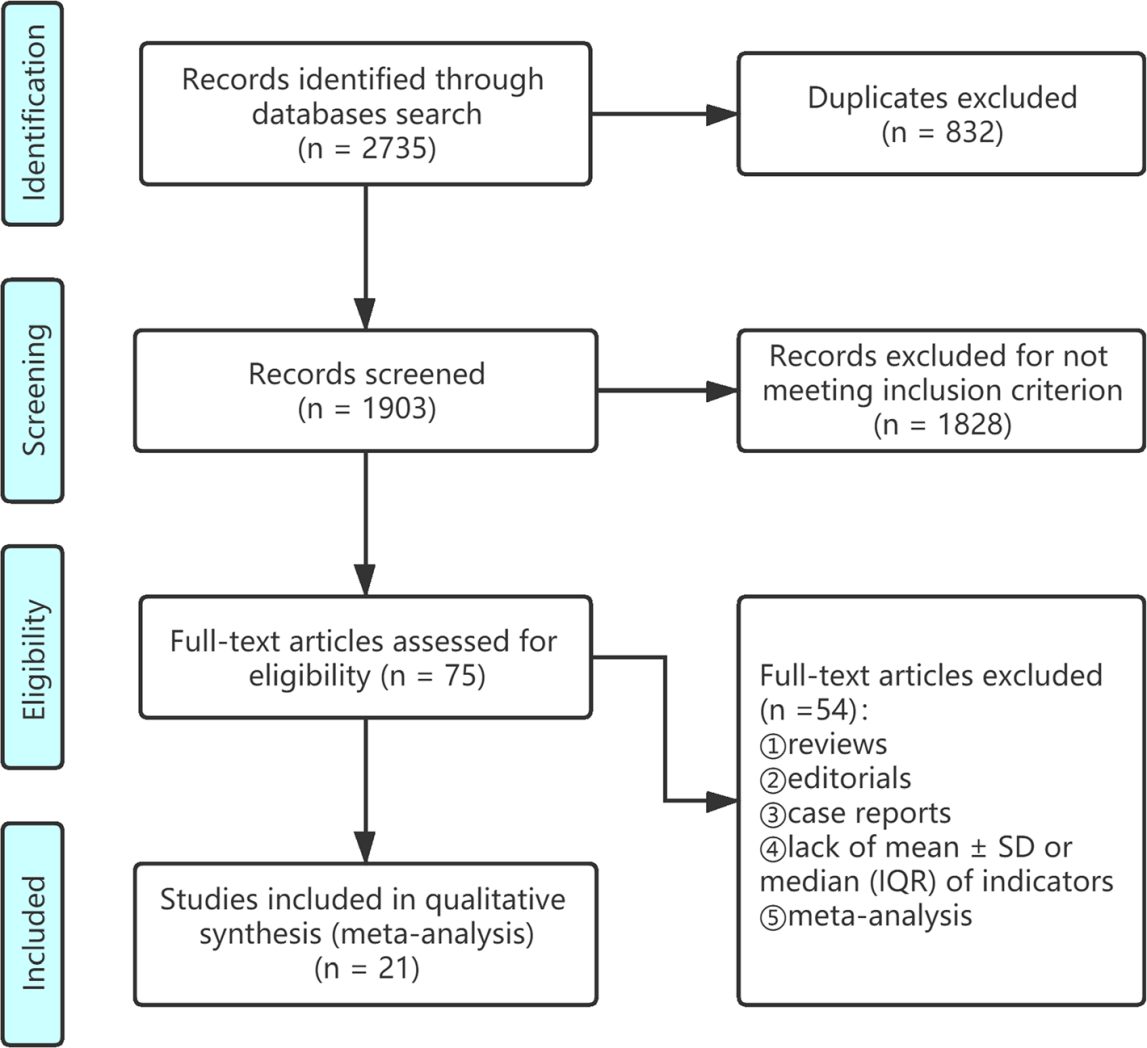
Flow diagram for studies selection

Figure 1 provides the flow diagram for study selection. Based on the inclusion criteria, 75 full articles were retrieved and 21 of these were included in the final meta-analysis. Duplicate publications, reviews, editorials, case reports, and studies without medians (IQR) and mean ± SD of indicators were excluded. Table 1 presents the characteristics of the 21 included studies, with 758 severe cases and 1275 mild cases of COVID-19. All but one prospective study [9] of the studies included in this meta-analysis were retrospective studies, which were mostly performed in China. All studies were deemed of high quality with NOS scores at 7 or above. Details can be found in Table 2.

**Table 1:** Characteristics of studies included in the meta-analysis

Author	Study Design	Country	Age (years)	Sample size (Severe)	Sample size (Mild)	Sample size	Sex (male %)
Chen G. 2020 [3]	Retrospective	China	56 (50–65)	11	10	21	17 (81%)
Chen R. 2020 [31]	Retrospective	China	56 ± 15	155	345	500	313 (57.1%)
Chi Y. 2020 [10]	Retrospective	China	46	8	22	30	/
Du R. 2020 [9]	Prospective	China	69 ± 8	21	42	63	30 (47.6%)
He R. 2020 [6]	Retrospective	China	49 (34–62)	69	135	204	79 (38.7%)
Jiang M. 2020 [12]	Retrospective	China	46 (17–88)	17	86	103	58 (56.3%)
Li S. 2020 [24]	Retrospective	China	46	26	43	69	40 (60.0%)
Liu Y. 2020 [32]	Retrospective	China	/	30	46	76	/
Ma J. 2020 [33]	Retrospective	China	62 (59–70)	17	20	37	20 (54%)
Pallotto C. 2020 [34]	Retrospective	Italy	65	13	25	38	19 (50.0%)
Qin C. 2020 [4]	Retrospective	China	58 (47–67)	27	17	44	235 (52%)
Sophie H. 2020 [5]	Retrospective	France	66	38	36	74	60 (81.1%)
Sun D. 2020 [15]	Retrospective	China	65	11	25	36	29 (80.5%)
Urra J.M. 2020 [35]	Retrospective	Spain	59	27	145	172	104 (60.5%)
Wan S. 2020 [13]	Retrospective	China	46	21	102	123	66 (53.7%)
Wang F. 2020 [18]	Retrospective	China	69 ± 9	14	14	28	21 (75%)
Zhang J. 2020 [19]	Retrospective	China	38 (32–57)	93	18	111	46 (41.4%)
Zhao Y. 2020 [7]	Retrospective	China	48	18	53	71	30 (42.3%)
Zheng Y. 2020 [36]	Retrospective	China	49	26	63	89	/
Zhou Y. 2020 [14]	Retrospective	China	42	5	12	17	6 (35.3%)
Zhu Z. 2020 [16]	Retrospective	China	51	111	16	127	45 (35.4%)

Age is described as mean or mean ± SD or median (IQR)

**Table 2:** Newcastle-Ottawa Scale (NOS)

Included studies	Is the definition adequate?	Represent activeness of the cases	Selection of controls	Definition of controls	Comparability of both groups	Ascertainment of diagnosis	Same ascertainment method for both groups	Nonresponse rate	Total scores
Chen G. 2020 [3]	☆	☆	☆	☆	☆	☆	☆	☆	8
Chen R. 2020 [31]	☆	☆	☆	☆	☆	☆	☆	☆	8
Chi Y. 2020 [10]	☆	☆	☆	☆	☆	☆	☆	☆	8
Du R. 2020 [9]	☆	☆	☆	☆	☆	☆	☆	☆	8
He R. 2020 [6]	☆	☆	☆	☆	☆	☆	☆	☆	8
Jiang M. 2020 [12]	☆	☆	☆	☆	☆	☆	☆	☆	8
Li S. 2020 [24]	☆	☆	☆	☆	☆☆	☆	☆	☆	9
Liu Y. 2020 [32]	☆	☆	☆	☆	/	☆	☆	☆	7
Ma J. 2020 [33]	☆	☆	☆	☆	☆☆	☆	☆	☆	9
Pallotto C. 2020 [34]	☆	☆	☆	☆	☆☆	☆	☆	☆	9
Qin C. 2020 [4]	☆	☆	☆	☆	☆	☆	☆	☆	8
Sophie H. 2020 [5]	☆	☆	☆	☆	☆☆	☆	☆	☆	9
Sun D. 2020 [15]	☆	☆	☆	☆	☆	☆	☆	☆	8
Urra J.M. 2020 [35]	☆	☆	☆	☆	☆☆	☆	☆	☆	9
Wan S. 2020 [13]	☆	☆	☆	☆	☆	☆	☆	☆	8
Wang F. 2020 [18]	☆	☆	☆	☆	☆☆	☆	☆	☆	9
Zhang J. 2020 [19]	☆	☆	☆	☆	☆	☆	☆	☆	9
Zhao Y. 2020 [7]	☆	☆	☆	☆	☆☆	☆	☆	☆	9
Zheng Y. 2020 [36]	☆	☆	☆	☆	☆	☆	☆	☆	8
Zhou Y. 2020 [14]	☆	☆	☆	☆	☆	☆	☆	☆	8
Zhu Z. 2020 [16]	☆	☆	☆	☆	☆	☆	☆	☆	8

### Associations of immune cells with COVID-19 severity

**Fig. 2:**
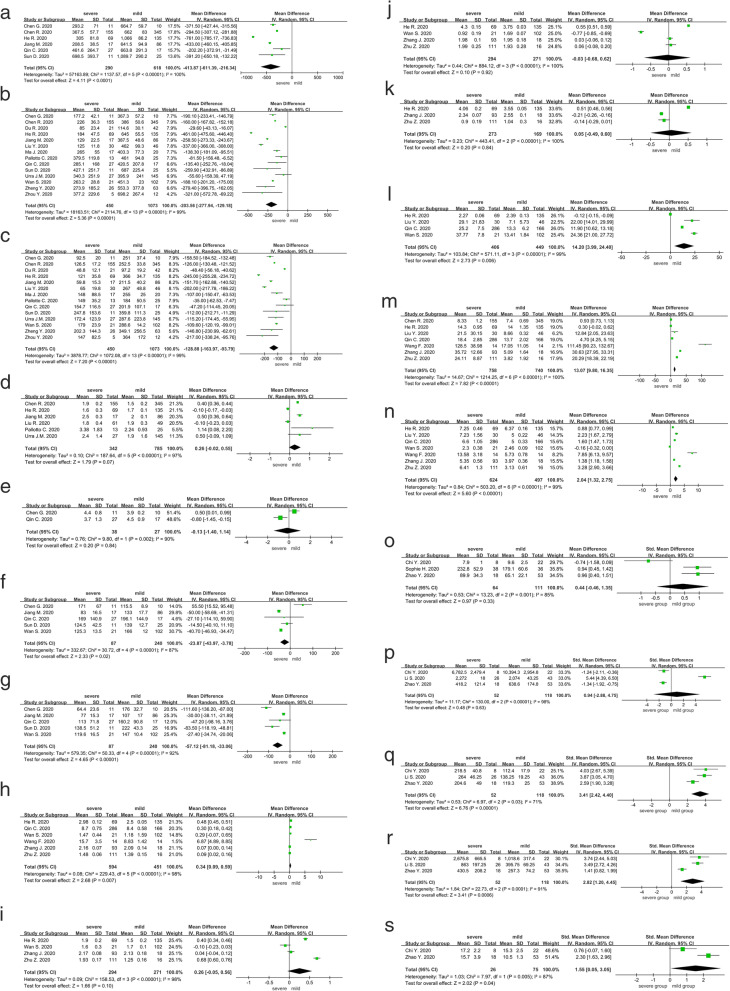
**a**. CD3+ T cell. **b**. CD4+ T cell. **c**. CD8+ T cell. **d**. CD4+ / CD8+ ratio. **e**. Treg cell. **f**. B cell. **g**. NK cell. **h**. TNF-a. **i**. IFN-y. **j**. IL-2. **k**. IL-4. **l**. IL-5. **m**. IL-6. **n**. IL-10. **o**. GM-CSF. **p**. RANTES. **q**. MCP-1. **r**. IP-10. 2 **s**. Eotaxin

Compared with mild cases, severe cases showed significantly lower levels of immune cells as CD3^+^ T cell (× 10^6^, MD, − 413.87; 95%CI, − 611.39 to − 216.34; *I*^*2*^, 100%; *p <* 0.001, Fig. 2a) with specifically CD4^+^ T cell (× 10^6^, MD, − 203.56; 95%CI, − 277.94 to − 129.18; *I*^*2*^, 99%; *p <* 0.001, Fig. 2b) and CD8^+^ T cell (× 10^6^, MD, − 128.88; 95%CI, − 163.97 to − 93.79; *I*^*2*^, 99%; *p <* 0.001, Fig. 2c), B cell (× 10^6^/L; MD, − 23.87; 95%CI, − 43.97 to − 3.78; *I*^*2*^, 87%; *p <* 0.001, Fig. 2f), and NK cell (× 10^6^/L; MD, − 57.12; 95%CI, − 81.18 to − 33.06; *I*^*2*^, 92%; *p <* 0.001, Fig. 2g). However, no significant difference was found in the other indicators as CD4^+^/CD8^+^ ratio (MD, 0.26; 95%CI, − 0.02 to 0.55; *I*^*2*^, 97%; *p <* 0.001, Fig. 2d) and Treg cell (× 10^6^, MD, − 0.13; 95%CI, − 1.40 to 1.14; *I*^*2*^, 90%; *p =* 0.002, Fig. 2e).

### Associations of cytokines with COVID-19 severity

Compared with mild cases, severe cases showed significantly higher levels of cytokines including TNF-α (pg/ml; MD, 0.34; 95%CI, 0.09 to 0.59; *I*^*2*^, 98%; *p <* 0.001, Fig. 2h), IL-5 (pg/ml; MD, 14.20; 95%CI, 3.99 to 24.4; *I*^*2*^, 99%; *p <* 0.001, Fig. 2l), IL-6 (pg/ml; MD, 13.07; 95%CI, 9.80 to 16.35; *I*^*2*^, 100%; *p <* 0.001, Fig. 2m), and IL-10 (pg/ml; MD, 2.04; 95%CI, 1.32 to 2.75; *I*^*2*^, 99%; *p <* 0.001, Fig. 2n). However, no significant difference was found in the other cytokines as IFN-γ (pg/ml; MD, 0.26; 95%CI, − 0.05 to 0.56; *I*^*2*^, 98%; *p <* 0.001, Fig. 2i), IL-2 (pg/ml; MD, 0.05; 95%CI, − 0.49 to 0.6; *I*^*2*^, 100%; *p <* 0.001, Fig. 2j), and IL-4 (pg/ml; MD, − 0.03; 95%CI, − 0.68 to 0.62; *I*^*2*^, 100%; *p <* 0.001, Fig. 2k).

### Associations of chemokines with COVID-19 severity

Compared with mild cases, severe cases showed significantly higher levels of chemokines including MCP-1 (SMD, 3.41; 95%CI, 2.42 to 4.40; *I*^*2*^, 71%; *p =* 0.03, Fig. 2q), IP-10 (SMD, 2.82; 95%CI, 1.20 to 4.45; *I*^*2*^, 91%; *p <* 0.001, Fig. 2r), and eotaxin (SMD, 1.55; 95%CI, 0.05 to 3.05; *I*^*2*^, 87%; *p =* 0.01, Fig. 2s). However, there was no significant difference in the other chemokines as GM-CSF (SMD, 0.44; 95%CI, − 0.46 to 1.35; *I*^*2*^, 85%; *p =* 0.001, Fig. 2o) and RANTES (SMD, 0.94; 95%CI, − 2.88 to 4.75; *I*^*2*^, 98%; *p <* 0.001, Fig. 2p).

### Sensitivity analysis

Significant heterogeneity was identified across all the comparisons (Fig. 2). Sensitivity analyses showed that our results were not obviously impacted by exclusion to any specific study for “CD3^+^ T”, “CD4^+^ T”, “CD8^+^ T”, “CD4^+^/CD8^+^”, “Treg”, “B cell”, “NK cell”, “TNF-α” “IFN”, “IL-2”, “IL-4”, “IL-5”, “IL-6”, “IL-10”, “RANTES”, “MCP-1”, “IP-10” and “eotaxin” between the severe group and the mild group. After excluding the study Chi et al. [10] on GM-CSF (SMD, 0.94; 95%CI, 0.58 to1.31), the sensitivity findings showed that there was a significant difference between pre- and post-sensitivity pooled SMDs, suggesting that it is better to keep this result in the meta-analysis. As such, our sensitivity analysis indicates that most of our results are reliable.

### Publication bias

**Fig. 3:**
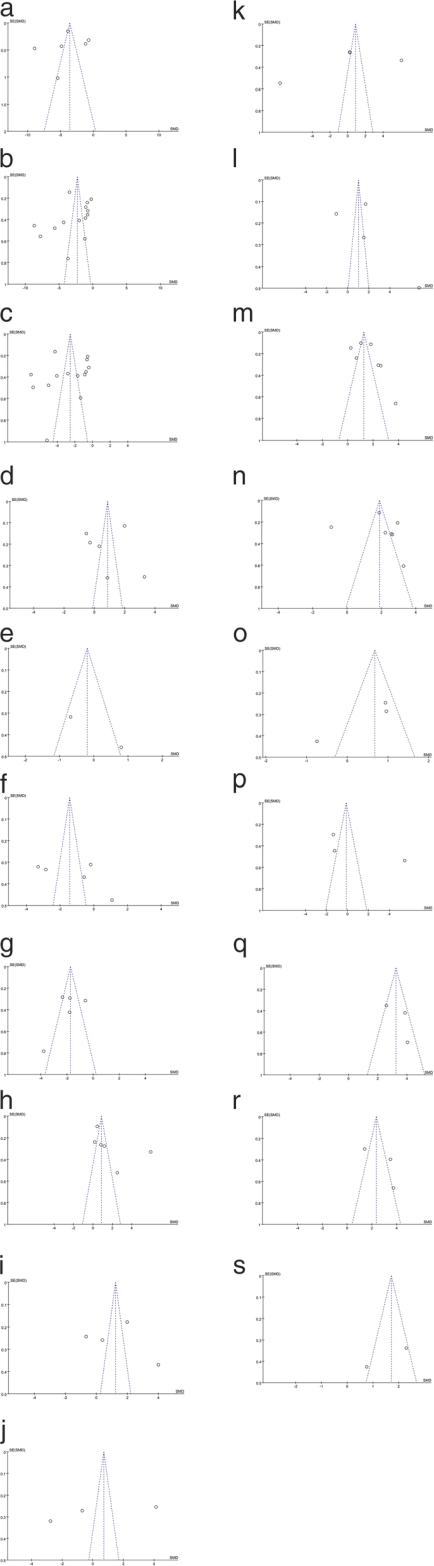
**a**. CD3+ T cell. **b**. CD4+ T cell. **c**. CD8+ T cell. **d**. CD4+ / CD8+ ratio. **e**. Treg cell. **f**. B cell. **g**. NK cell. **h**. TNF-a. **i**. IFN-y. **j**. IL-2. **k**. IL-4. **l**. IL-5. **m**. IL-6. **n**. IL-10. **o**. GM-CSF. **p**. RANTES. **q**. MCP-1. **r**. IP-10. **s**. Eotaxin

We assessed the publication bias of the literature by means of the funnel plots in all included studies of each indicator. Funnel plot analysis did not detect obvious publication bias as the shape of all funnel plots did not reveal any evidence of obvious asymmetry (Fig. 3).

## Discussion

It is necessary to investigate the host immune response to SARS-CoV-2, which may help identify immune markers of disease severity for effective triage of COVID-19 patients [11]. Our study compared the levels of immune cells, cytokines and chemokines between mild and severe COVID-19 patients.

The variations of immune cell levels are inconsistent among different reports. The majority of our included studies reported significantly lower levels of immune cells (CD3^+^ T, CD8^+^ T, CD4^+^ T, B and NK cells) in severe cases compared with mild cases [3, 12, 13]. Only two studies didn’t find significant reductions in CD8^+^ T cells [4, 14], while one study reported an increase of B cell [15] in severe cases. Using all the collected evidence, our meta-analysis results found that the decrease in CD3^+^ T, CD8+ T, CD4+ T, B and NK cells was significant in severe cases, but Treg cells and the ratio of CD4^+^/CD8^+^ T cells showed no significant difference.

The mechanism underlying the reduction of immune cell levels and COVID-19 severity remains to be determined. CD8^+^ T cells exert their effects mainly through two mechanisms, including cytolytic activity against target cells and cytokine secretion [16]. CD4^+^ T cells are capable of activating the CD8^+^ T cell response to acute respiratory virus infection [11]. SARS-CoV-2 and associated autoimmune antibodies may contribute to the growth inhibition and apoptosis of immune cells [6, 17].

The cytokine variations of various studies are also inconsistent. Except for one study on IL-6 [6] and another study on TNF-α, most of our included studies reported that IL-6 and TNF-α levels were significantly higher in severe cases compared with mild cases [4, 16, 18, 19]. Some of our included studies found no significant difference in the levels of IL-2, IL-4, IL-5, and IFN-γ, while almost an equivalent number of studies of each indicator found that they were significantly higher in severe cases. Synthesizing all the collected evidence, our meta-analysis results found that IL-5, IL-6, IL-10 and TNF-α levels were significantly higher in severe cases compared with mild cases. However, the levels of IL-2, IL-4, IFN-γ, Treg cells and CD4^+^/CD8^+^ ratios showed no significant differences.

In severely infected individuals, SARS-CoV-2 could induce an excessive cytokine response, such as IL-6, IL-10, and TNF-α surge, known as cytokine storm. Cytokine storms may contribute to acute respiratory distress syndrome (ARDS) or multiple-organ dysfunction, resulting in physiological deterioration and death [20]. Cytokines such as IL-10, IL-6, and TNF-α are also involved in the decline of T cell counts. IL-6 contributes to host defense by stimulating acute phase responses [21]. TNF-α is a pro-inflammatory cytokine that can promote T cell apoptosis [22]. Patients requiring ICU admission have significantly higher levels of IL-6, IL-10, and TNF-α. Further, the levels of IL-6, IL-10, and TNF-α inversely correlate with CD4^+^ and CD8^+^ T cell counts [23]. This fact is strengthened by our meta-analysis results.

SARS-CoV-2 infection is a potent inducer of proinflammatory chemokines that are potentially involved in the defense against viral infection [10]. Some studies have reported higher concentrations of GM-CSF [5], IP-10 [5, 10, 24], MCP-1 [10, 24], eotaxin [7] and RANTES [24] between severe cases and mild cases. However, other studies have not revealed significant differences in the concentrations of GM-CSF [7, 10], IP-10 [7], RANTES [10], MCP-1 [7], and eotaxin [10] . Synthesizing all the collected evidence, our meta-analysis results found that MCP-1, IP-10 and eotaxin levels were significantly higher in severe cases compared with mild cases. However, levels of GM-CSF and RANTES showed no significant differences. Binding to the chemokine receptor 3, IP-10 activates and recruits leucocytes, including T cells and monocytes [25]. MCP-1-mediated migration of monocytes from the blood stream through the vascular endothelium is essential for routine immune surveillance in tissues in response to inflammation [26]. Abnormally elevated MCP-1, IP-10 and eotaxin levels may help to determine the severity of SARS-CoV-2 infections and serve as prognostic markers for disease progression.

Cytokines and chemokines play a key role in the pathogenesis of ARDS. After SARS-CoV-2 infection, vascular endothelial cells become dysfunctional and IL-6, TNF-a, and MCP-1 levels are elevated, leading to COVID-19-associated vascular inflammation and coagulopathy, particularly endotheliitis in the lungs, heart and kidney [27, 28]. TNF-a is central in the pathogenesis of inflammation and triggers the release of many inflammatory mediators including IL-1, IL-6, IL-8, and GM-CSF [29]. TNF-a can also disintegrate the endothelial and epithelial cytoskeleton, resulting in alveolar-capillary barrier disruption, vascular leakage and alveolar edema, which in turn leads to hypoxia in the body [30].

### Limitations

Several limitations of our study should be considered. Firstly, the number of studies and participants was not large enough for publication bias analysis of most indicators. Secondly, the majority of the included studies in this meta-analysis were retrospectives. Thirdly, the overall generalizability of the meta-analysis results should be interpreted with caution as most of the included studies were conducted in China due to limitations in geographic distribution and ethnic diversity. It would be better to include more studies with a broad geographic scope to gain a more comprehensive understanding of the immunological features of COVID-19 patients.

## Conclusions

Our synthesized results revealed significantly lower levels of immune cells in CD3^+^ T, CD4^+^ T, CD8^+^ T, B and NK cells, higher levels of cytokines (TNF-α, IL-5, IL-6 and IL-10) and higher levels of chemokines (MCP-1, IP-10 and eotaxin) in severe cases compared with mild cases of COVID-19. However, there was no significant difference in levels of Treg cell, the ratio of CD4^+^/CD8^+^ T cell, IL-2, IL-4, IFN-γ, GM-CSF and RANTES. Measurement of immune cells and cytokines may help identify immune markers of COVID-19 severity and contribute to the development of immunologic therapies and vaccines.

## Supplementary Information

**Additional file 1.**

## Data Availability

The datasets used and/or analysed during the current study are available from the corresponding author on reasonable request.

## Abbreviations

COVID-19: Coronavirus disease 2019
SARS-CoV-2: Severe acute respiratory syndrome coronavirus 2
MD: Mean difference
IQR: Interquartile range
IL-2: Interleukin-2
IL-4: Interleukin-4
IL-5: Interleukin-5
IL-6: Interleukin-6
IL-10: Interleukin-10
TNF-α: Tumor necrosis factor alpha
IFN-γ: Interferon gamma
NK cell: Natural killer cell
CI: Confidence interval
NOS: Newcastle-ottawa scale
ARDS: Acute respiratory distress syndrome
SD: Standard deviation
GM-CSF: Granulocyte-macrophage colony-stimulating factor
RANTES (CCL5): Regulated upon activation, normal T-cell expressed and secreted
IP-10 (CXCL10): IFN-γ Inducible protein-10
MCP-1 (CCL2): Monocyte chemoattractant protein-1
